# Multi-method Examination of Elder Mistreatment in the Age of COVID-19

**DOI:** 10.1093/geroni/igab046.2836

**Published:** 2021-12-17

**Authors:** Sonia Salari, Sharon Talboys, Annie Isabel Fukushima, Heather Melton, Seage Michelle, Megan Petersen

**Affiliations:** 1 University of Utah, Salt Lake City, Utah, United States; 2 University of Utah, University of Utah, Utah, United States

## Abstract

A multi-method study exposed COVID-19 influence on the pre-existing epidemic of elder mistreatment in Utah. We found changes in 1) abuse types, 2) service responses, 3) firearm access and 4) policy implications. Gun sales were tracked by news surveillance and FBI National Instant Criminal Background Check System (NICS) for pre-pandemic (2018/2019) and pandemic years (2020/2021). New requests for permits skyrocketed during the pandemic. The 2021 Utah State Legislature loosened restrictions on concealed permits. Domestic violence (DV) Fatality Tracker Data in pre-covid years were compared to 2020-2021. A figure illustrates the prevalence of DV fatalities, ages of victims by year and methods used. We conducted 15 in-depth interviews of stake holders who serve DV victims (shelters, police, etc.). DV shelters had a relative lack of children during the pandemic, but increased use by older persons 60+. Susceptibility to chronic respiratory distress syndrome, required social distance for older persons. DV shelters obtained CARES Act funds to adapt solutions, like placing victims in hotel rooms. Most victims stayed at home, confined with abuser(s), some without technology, so isolation decreased their safety. Evidence suggests some fatalities among elder adults. A case study during the pandemic described a 73-year-old mother’s suspicious bank account activity. Bank employees sent police to her home. She was missing, but her co-resident adult son was in possession of her bank cards. She was later found in a shallow grave. Utah households have increased risks of DV fatalities in the wake of the pandemic and for years to come.

